# Sharp loss: a new loss function for radiotherapy dose prediction based on fully convolutional networks

**DOI:** 10.1186/s12938-021-00937-w

**Published:** 2021-10-09

**Authors:** Xue Bai, Jie Zhang, Binbing Wang, Shengye Wang, Yida Xiang, Qing Hou

**Affiliations:** 1 Department of Radiation Physics, Zhejiang Key Laboratory of radiation Oncology, The Cancer Hospital of the University of Chinese Academy of Sciences (Zhejiang Cancer Hospital), Hangzhou, 310022 China; 2 Key Laboratory of Radiation Physics and Technology, Ministry of Education, Institute of Nuclear Science and Technology, Sichuan University, Chengdu, 610064 China; 3 School of Nuclear Science and Technology, University of South China, Hengyang, 421000 China

**Keywords:** Radiotherapy, Dose prediction, Loss function, Breast cancer

## Abstract

**Background:**

Neural-network methods have been widely used for the prediction of dose distributions in radiotherapy. However, the prediction accuracy of existing methods may be degraded by the problem of dose imbalance. In this work, a new loss function is proposed to alleviate the dose imbalance and achieve more accurate prediction results. The U-Net architecture was employed to build a prediction model. Our study involved a total of 110 patients with left-breast cancer, who were previously treated by volumetric-modulated arc radiotherapy. The patient dataset was divided into training and test subsets of 100 and 10 cases, respectively. We proposed a novel ‘sharp loss’ function, and a parameter *γ* was used to adjust the loss properties. The mean square error (MSE) loss and the sharp loss with different *γ* values were tested and compared using the Wilcoxon signed-rank test.

**Results:**

The sharp loss achieved superior dose prediction results compared to those of the MSE loss. The best performance with the MSE loss and the sharp loss was obtained when the parameter *γ* was set to 100. Specifically, the mean absolute difference values for the planning target volume were 318.87 ± 30.23 for the MSE loss versus 144.15 ± 16.27 for the sharp loss with *γ* = 100 (*p* < 0.05). The corresponding values for the ipsilateral lung, the heart, the contralateral lung, and the spinal cord were 278.99 ± 51.68 versus 198.75 ± 61.38 (*p* < 0.05), 216.99 ± 44.13 versus 144.86 ± 43.98 (*p* < 0.05), 125.96 ± 66.76 versus 111.86 ± 47.19 (*p* > 0.05), and 194.30 ± 14.51 versus 168.58 ± 25.97 (*p* < 0.05), respectively.

**Conclusions:**

The sharp loss function could significantly improve the accuracy of radiotherapy dose prediction.

## Background

One of the goals of radiotherapy planning is to protect normal tissue as much as possible while delivering sufficient doses to the tumor. Because different organs have different sensitivity to radiation, their dose constraints are also different. A key problem in external radiotherapy planning is to judge whether the selected plan achieves the optimal dose distribution while minimizing the adverse effects on the organs at risk (OAR). In precision radiotherapy, personalized schemes should be established for evaluating the planning quality. Automated radiotherapy planning and quality control have been commonly based on integrating and summarizing historical data of expert-level treatment plans as well as building models to predict reasonable and achievable dosimetric indicators for new cases [1, 2]. Numerous models have been developed for predicting achievable OAR constraints [2–9] and dose–volume histograms (DVH) [10–12].

Because a dose distribution is generally a better descriptor of radio-therapeutic prognosis than a single DVH index, more recently, dose distribution prediction from computed tomography (CT) images and regions of interest (ROI) has been the focus of several studies [13–24]. Also, deep learning methods have been recently proposed for predicting 3D dose distributions. Prominent examples of these methods utilize enhanced variants of the traditional convolutional neural networks (CNN), namely, the fully convolutional networks (FCN). A FCN is obtained through replacing the last fully connected layer in a CNN by a convolutional layer. The FCN architecture has been already used for medical image segmentation [25–28], and it has demonstrated outstanding performance in radiotherapy dose prediction for a variety of cancer types, including nasopharyngeal cancer, lung cancer, prostate cancer and breast cancer [16, 19–23].

In radiotherapy, the region of clinical concern usually accounts for only a small part of the whole imaged region. This data imbalance can lead to a decrease in the accuracy of a dose prediction model, because the model would then tend to make dose predictions closely matching the dosage values for the clinically irrelevant regions. The problem of data imbalance in deep learning has been previously addressed and successfully alleviated through special loss functions such as the focal loss designed for dense object detection and the dice loss employed in natural language processing tasks [29, 30]. Further studies have been conducted to boost the dose prediction accuracy in radiotherapy by network structure adjustment, network parameter optimization and adding beam influence factors to network inputs [16, 19–21]. However, loss function design for dose prediction in radiotherapy has been generally overlooked. Inspired by earlier performance improvements based on the focal and dice loss functions, we propose a novel ‘sharp loss’ function for better handling of data imbalance problems, and incorporate this loss in an FCN framework for dose prediction in radiotherapy. The sharp loss is a dynamically scaled variant of the classical mean-squared-error (MSE) loss, where the scaling factor decreases in low-dose regions. The effectiveness of this proposed loss function is demonstrated using a U-Net architecture for dose distribution prediction in radiotherapy of left-sided breast cancer.

## Results

**Fig. 1:**
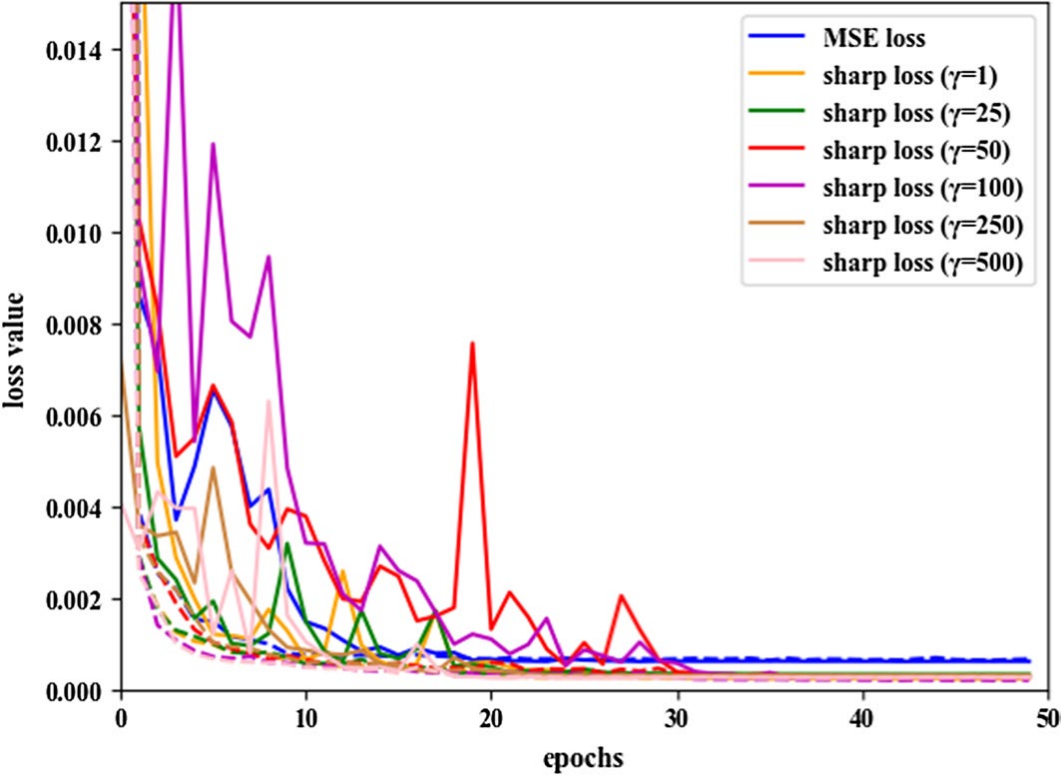
Dose prediction performance with different loss functions: the training loss (dotted) and validation loss (solid) curves for the MSE loss and the sharp loss with different values of *γ*

Figure 1 shows the training and validation loss curves for the MSE loss and the sharp loss with different values of $\gamma$. The U-Net architecture equipped with the sharp loss achieved smaller loss than the same architecture with the MSE loss. In the first 30 epochs, the validation loss with the sharp loss function and factors of $\gamma = 50$ and $\gamma = 100$ show more variations between the cross-validation folds than those of the other loss function variants. However, all the training and validation loss curves tended to be flat after 32 epochs. This indicates that the U-Net model has good generalization for dose prediction.

Table 1 compares the mean absolute difference (MAD) values of different dose regions in the test data for the MSE loss and the sharp loss with different values of *γ*. Clearly, dose prediction results based on the MSE loss achieve small MAD values in all regions, but the low-error voxels are concentrated outside the body. Inside the body, the MAD values are large for high ground-truth dose values. The standard deviation (std) explain the performance of the prediction model in different dose area. The smaller the std, the better the generalization performance of the model in different dose regions. The std of the prediction error inside the body was 312.57 cGy with the MSE loss. This standard deviation becomes smaller when the sharp loss is used with *γ* = 1, 25, 50 or 100. In particular, the standard deviation has the smallest value for *γ* = 100. For *γ* < 100, the prediction precision inside the body increases as the value of *γ* becomes larger, and the sharp loss prediction results were better than those of the MSE loss. However, when *γ* > 100, the precision deteriorates again as the value of γ increased.

**Table 1:** The MAD values (cGy) of different dose regions in the test data for the MSE and sharp loss functions

	Loss function							Average dose gradient (cGy/mm)
	MSE loss	Sharp loss						
		*γ* = 1	*γ* = 25	*γ* = 50	*γ* = 100	*γ* = 250	*γ* = 500	
Whole region	56.72	43.78	52.45	43.40	84.57	690.74	950.98	–
Outside the body	32.93	24.31	34.31	24.10	71.82	765.24	1047.12	–
Inside the body	186.55	150.02	151.46	148.67	154.15	284.10	426.26	–
Region 0-500 cGy	109.43	87.46	99.06	99.68	110.40	265.66	457.31	1.97
Region 500–1000 cGy	294.03	262.00	228.30	220.12	221.34	245.57	224.44	78.67
Region 1000-2000 cGy	471.94	419.89	383.34	369.57	391.04	413.08	374.86	151.24
Region 2000–3000 cGy	649.82	530.12	504.69	472.01	488.17	517.25	485.96	267.06
Region 3000–4000 cGy	754.65	547.15	521.36	491.88	459.56	489.77	474.44	330.70
Region 4000–5000 cGy	645.23	453.75	407.67	410.46	319.72	410.30	379.45	222.84
Region > 5000 cGy	336.25	241.70	214.88	193.21	151.32	249.87	229.58	48.69
Std	312.57	254.86	245.41	236.20	228.79	374.80	555.69	-

The last line shows the standard deviation values of the respective prediction errors inside the body. The last column shows the average dose gradient of the respective dose region in ground-truth

Table 2 shows the MAD values of different ROI types for the MSE and sharp loss functions. The sharp loss function with *γ* = 100 shows the best performance for the planning target volumes (PTV), the ipsilateral lung, the heart and the contralateral lung. For the spinal cord, similar prediction errors are obtained using the sharp loss function with *γ* = 1, 25, 50 and 100. These errors are lower than those resulting from other loss variants.

**Table 2:** The MAD values (cGy) for each ROI type in the test data for the MSE and sharp loss functions

	Loss function						
	MSE loss	Sharp loss					
		*γ* = 1	*γ* = 25	*γ* = 50	*γ* = 100	*γ* = 250	*γ* = 500
PTV	316.49	229.15	201.06	182.38	139.85	239.11	222.68
Ipsilateral lung	271.81	232.06	215.22	215.47	189.99	263.82	362.17
Heart	218.28	185.42	183.79	175.73	144.41	217.63	218.69
Contralateral lung	125.96	116.21	117.38	136.70	113.42	172.97	241.52
Spinal cord	195.51	162.50	168.47	160.04	170.41	228.89	310.26

Table 3 shows the MAD values for different ROI types of the test data, along with statistical significance measures of the differences between the results obtained with the MSE loss and the sharp loss with *γ* = 100. Significant statistical differences were found for all ROI types, except for the case of the contralateral lung. The results demonstrated that using the sharp loss (*γ* = 100) leads to superior dose prediction results in comparison to the MSE loss.

**Table 3:** The MAD values (cGy) for different ROI types and the p-values of the statistical differences between the testing results obtained with the MSE and sharp loss functions

	MSE loss	Sharp loss (γ = 100)	p
PTV	318.87 ± 30.23	144.15 ± 16.27	0.005
Ipsilateral lung	278.99 ± 51.68	198.75 ± 61.38	0.005
Heart	216.99 ± 44.13	144.86 ± 43.98	0.005
Contralateral lung	125.96 ± 66.76	111.86 ± 47.19	0.074
Spinal cord	194.30 ± 14.51	168.58 ± 25.97	0.005

**Fig. 2:**
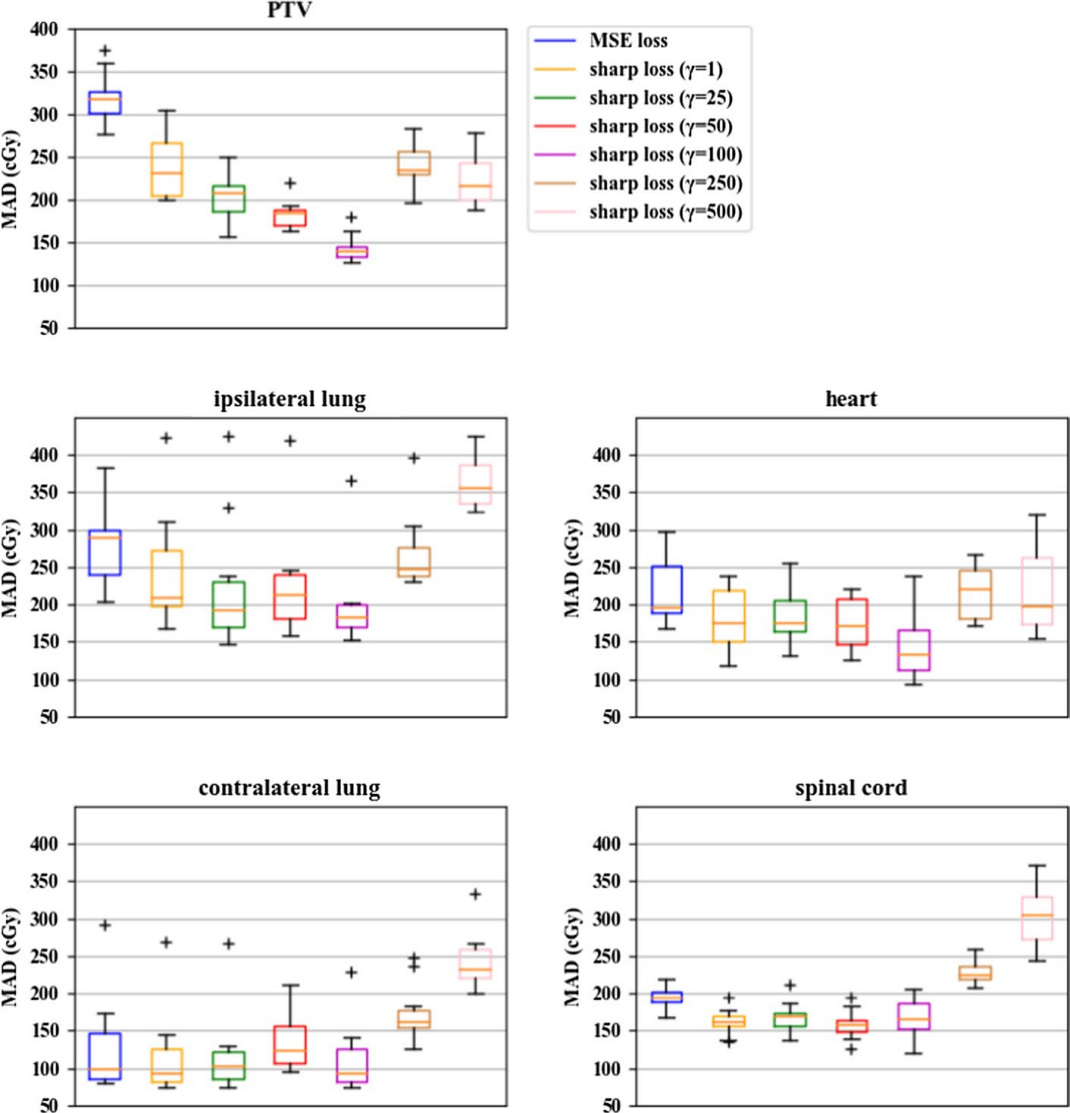
Boxplots of the MAD values obtained for each ROI type of the test data

Figure 2 shows boxplots of the MAD values obtained for each ROI type of the test data. This figure indicates that the prediction results obtained with the sharp loss at *γ* = 100 are more accurate and stable than the results obtained by other loss variants.

## Discussion

**Fig. 3:**
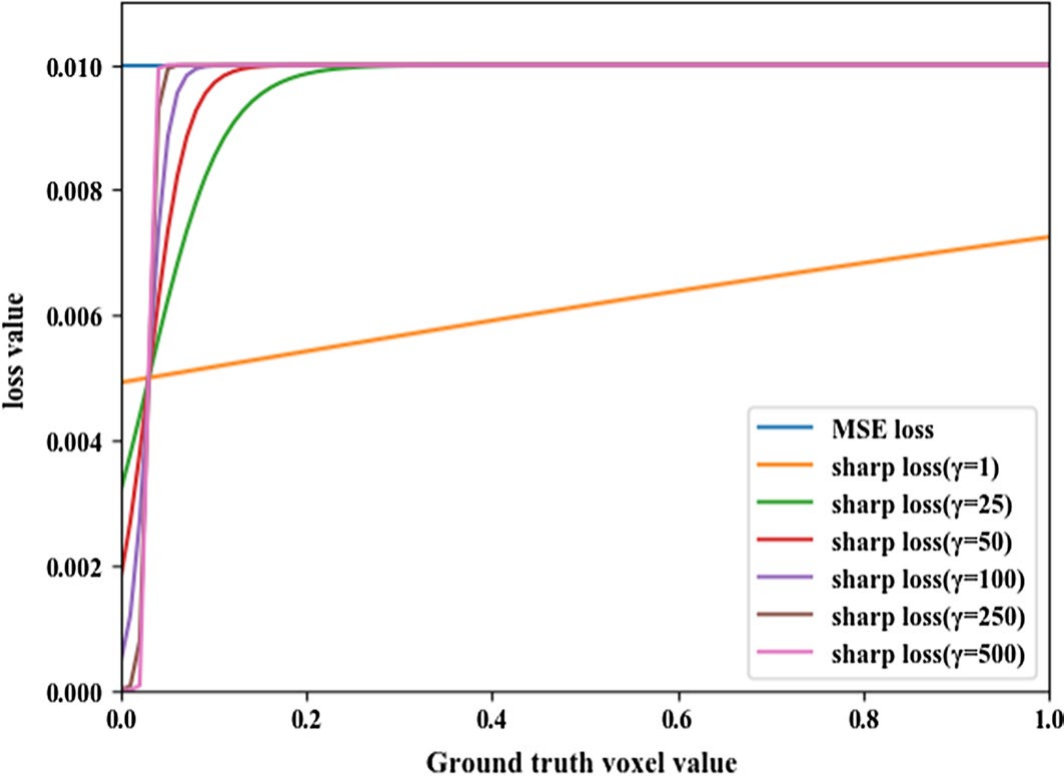
The MSE loss function and the sharp loss function for *γ* = 1, 25, 50, 100, 250, and 500. For a fixed error of 0.1 in the predicted dose value, the loss value changed for different variants of the sharp loss function in the low-dose area. A voxel value 1.0 corresponds to 6000 cGy

This study demonstrated the effectiveness of the proposed sharp loss function for dose prediction in radiotherapy. As Fig. 3 shows, the sharp loss function can be divided into two stages. A ground-truth value $D_{0}$ was defined as the demarcation point of these two stages, i.e.,

1 $$\begin{gathered} {\text{when}}\;D_{i} = D_{0} , \hfill \\ {\text{sharp loss}}\;(D_{i} ) = 0.99 \times {\text{MSE loss}}{.} \hfill \\ \end{gathered}$$

When $D_{i} \left[ {0,D_{0} } \right)$, the value of the sharp loss decreases with the decrease in dose. When $D_{i} \left[ {D_{0} ,6000} \right]$, the value of the sharp loss is approximately equal to the MSE loss. The γ factor affects the loss function in two ways. First, this factor influences the value of $D_{0}$. Second, the *γ* factor affects the slope in the range $\left[ {0,D_{0} } \right)$. As the *γ* value increases, the loss of prediction error decreases in low-dose regions.

The *γ* factor was experimentally fine-tuned and its best value was found to be 100. When γ = 100, $D_{0}$ = 455.71 cGy, which means that the sharp loss function would enable the U-Net model to pay more attention to the regions where the dose is larger than 455.71 cGy. With *γ* = 100 and a ground-truth dose $Dp_{i} = 0$, the sharp loss = 0.047 the MSE loss. When *γ* < 100, the $D_{0}$ value becomes larger than 455.71 cGy, and hence imprecise prediction results may be obtained in the region where the ground-truth dose values fall between 455.71 cGy and $D_{0}$. When *γ* > 100, loss values close to 0 would decrease quickly, and this can lead to imprecise prediction results in the region where the ground-truth dose values are between 0 and 455.71 cGy. This phenomenon can be observed in Table 1. The increase of γ reduces the dose prediction accuracy outside the body, but improves the model performance inside the body, especially in the region where the dose is larger than 3000 cGy. However, this effect is not obvious when *γ* is larger than 100. Since the whole region inside the body is of clinical concern when making a treatment plan and deserves the same level of attention for dose prediction, the standard deviation of the prediction error inside the body was calculated for each loss. The standard deviation has the lowest value when γ = 100. This means that paying sufficient attention to various dose regions inside the body is warranted to ensure balance in the U-Net optimization process.

Table 2 further illustrates the advantages of the proposed sharp loss with *γ* = 100. For most of the considered organs, this loss achieved the best dose prediction performance. Even though the sharp loss function with *γ* = 100 shows the lowest standard deviation of the MAD prediction error, the prediction results still vary across different regions. The largest MAD values occur in the regions where the dose values are between 2000 and 3000 cGy, while the smallest MAD values are observed in the regions with dose values smaller than 500 cGy or larger than 5000 cGy. These results can be ascribed to two reasons. First, the predicted results are prone to higher errors in regions with high dose gradients. Those regions usually have doses between 2000 and 5000 cGy. The average dose gradient is shown in the last column of Table 2. Second, a patient case can be associated with different expertise dose distributions. Therefore, even when the predicted dose values satisfy the optimal solution, there could still be some differences between the predicted and ground-truth dose values.

**Fig. 4:**
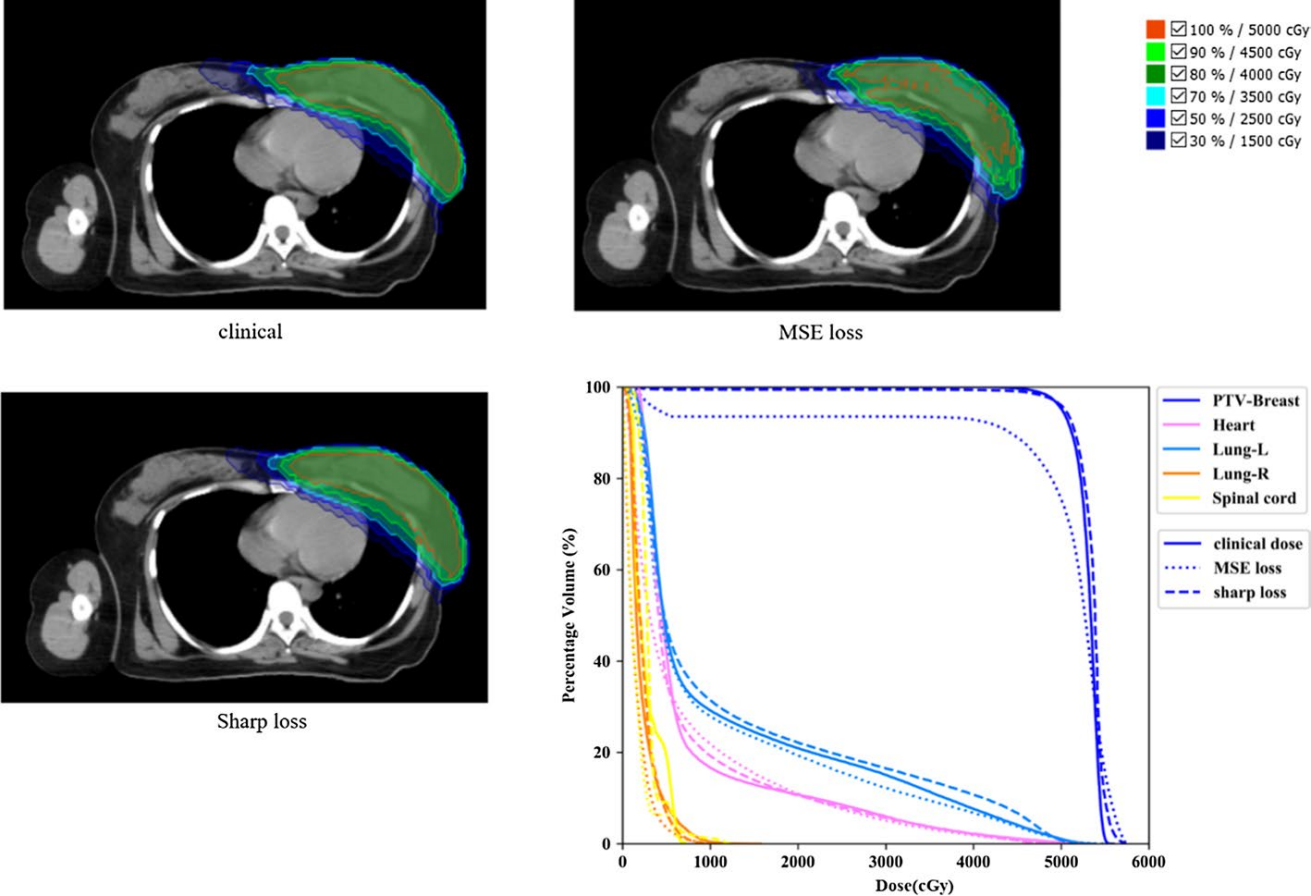
Comparison of the ground-truth and predicted dose distributions, with corresponding DVH, for the MSE loss and the sharp loss with *γ* = 100

Figure 4 presents a sample comparison of the clinical ground-truth dose distribution and the predicted dose distributions for the MSE and sharp loss functions, with the corresponding DVH. In this case, the prediction result using MSE loss shows a large error in the PTV region. There is a dose deficiency at the margin of PTV, and the predicted doses of some PTV voxels adjacent to the air are close to zero. Obviously, the sharp loss improves the dose distribution prediction in the ROI of the PTV. As discussed above, the sharp loss has a better correction in the areas with large dose gradients, while tumors near the body surface has a great dose drop at the air boundary. The sharp loss may bring greater improvement to the cases that the PTV near the body surface.

To provide a more realistic and intuitive estimate of the dosimetry, clinically relevant metrics are shown in Table 4. The homogeneity index (HI) is defined as $\left( {D_{1} - D_{99} } \right)/5000cGy$, and the conformation index (CI) is defined as $\left( {V_{{{\text{PTV}}}} \cap V_{{5000{\text{cGy}}}} } \right)^{2} /\left( {V_{{{\text{PTV}}}} \times V_{{5000{\text{cGy}}}} } \right)$. The clinical metrics of prediction results obtained with the sharp loss at *γ* = 100 are more accurate than the traditional MSE loss.

**Table 4:** The clinical metrics of ground-truth and predicted dose distribution using MSE loss and sharp loss (γ = 100)

	Ground truth	Predicted dose	
		MSE loss	Sharp loss (*γ* = 100)
PTV			
D95 (cGy)	5000.0 ± 0.0	4795.09 ± 173.60	4963 ± 96.30
HI	0.12 ± 0.03	0.42 ± 0.04	0.19 ± 0.02
CI	0.83 ± 0.04	0.56 ± 0.08	0.77 ± 0.63
Heart			
Mean (cGy)	625.42 ± 125.20	538.19 ± 147.21	644.46 ± 143.68
V30 (%)	4.73 ± 1.94	3.0 ± 1.77	4.03 ± 1.77
Ipsilateral lung			
Mean (cGy)	1065.04 ± 46.28	978.22 ± 73.49	1094.99 ± 125.57
V5 (%)	42.63 ± 2.73	44.8 ± 2.53	45.57 ± 4.13
V20 (%)	19.21 ± 1.18	17.02 ± 1.99	18.82 ± 3.12
V30 (%)	13.34 ± 1.28	9.76 ± 1.78	12.99 ± 2.60
Whole lung			
Mean (cGy)	597.11 ± 67.89	545.82 ± 52.60	623.70 ± 87.46
V5 (%)	24.31 ± 5.90	23.22 ± 2.31	22.94 ± 3.45
V20 (%)	9.02 ± 1.04	7.99 ± 1.12	8.87 ± 1.83
V30 (%)	6.28 ± 0.86	4.61 ± 0.81	6.15 ± 1.48

The FCN is widely used in radiotherapy dose prediction at present. Kajikawa used CNN-based methods to predict radiotherapy dose of prostate cancer [22]. Chen found that ResNet101 performed better than VGGNet16 in nasopharyngeal cancer [19]. Several studies used the U-Net, which is characterized by U-shaped architecture and up-sampling and down-sampling operations, to train small size medical dataset efficiently [20, 23]. Babier developed a 3D generative adversarial network for dose prediction and demonstrated superior performance on oropharyngeal cancer cases than previous approaches [31]. The above studies enhance the model performance by improving the network structure. Besides, Barragán-Montero improved the prediction results by adding beam configuration into the input data [21]. However, the above methods are not targeted to solve the problems caused by data imbalance.

Data imbalance is a key obstacle that deteriorates performance in tasks of dense object detection or semantic segmentation. Small organ segmentation in medical imaging is particularly quite challenging due to the large imbalance between the object and background classes [32, 33]. Such imbalance also emerges in dose prediction tasks in radiotherapy. Among all voxels of the 10 test cases of this study, about 74.52% of the voxels were outside the body and the corresponding dose values were just 0. Table 1 shows that the MAD value outside the body was much lower than that inside the body (32.93 versus 186.55 cGy) when the MSE loss function was used. This indicates that a large number of the voxels outside the body overwhelms the region inside the body. Even inside the patient body, the voxels whose dose values are lower than 500 cGy accounted for 77.30% of the total voxels. As a result, the MAD value for the region with the dose range 0–500 cGy is the lowest among all regions inside the body, and the high-dose region was ignored. Data augmentation is a common method to alleviate the effects of data imbalance in deep learning. An augmentation algorithm applies multiple types of transformations to the training data in order to increase the proportion of the samples for the weakly represented class. However, data augmentation is only suitable for cases where the input images and the associated ground-truth data are scale-independent, but dose distributions in radiotherapy are dependent on the X-ray penetration depth. Loss function redesign is another way to deal with the problem of data imbalance, and this has been validated by previous studies [29, 30]. In this study, the sharp loss was proposed to solve the data imbalance problem, and this loss has indeed demonstrated improved prediction performance. To the best of the authors’ knowledge, this is the first study that uses such novel loss function for improving the 3D dose prediction performance.

Nevertheless, this study has several limitations. First, the proposed sharp loss function was applied in conjunction with the U-Net architecture only and the data were limited to breast cancer cases. The next phase of this work will involve more types of deep networks as well as cancer types associated with other sites. Second, the recommended sharp loss factor, *γ* = 100, was selected based only on a limited validation dataset. With different networks or datasets, alternate suitable values of γ could be sought. Because of the different characteristics of radiotherapy planning for different human organs, the universality of the proposed sharp loss function will be investigated and scrutinized in future work. Third, the model was tested on 10 cases, which limited the evaluation of trained model generalization. The dataset will be expanded in the future. Furthermore, since one of the important applications of dose prediction is automatic planning, the related automatic plan research would be conducted in future work.

## Conclusions

This study proposes a novel loss function, named sharp loss, for FCN-based dose distribution prediction in radiotherapy. This loss function was first used to improve the outcomes of dose prediction modeling. The sharp loss function has achieved better prediction accuracy than that of the traditional MSE loss. With different values of the non-negative parameter γ greater than or equal to one, the value *γ* = 100 exhibited prediction results that are superior to those obtained with other values. In addition, the same γ value reduced the imbalance-induced prediction errors in regions of different doses. The sharp loss function could enable the prediction model to focus on the regions of more clinical significance. The improved predictions of dose distributions provide an effective foundation for quality assurance, automation, and efficiency in planning radiotherapy.

## Methods

### Dataset description

In this study, 110 patients in the early stages (Stage I and Stage II) of left-sided breast cancer were involved retrospectively. These patients received radiotherapy in the Zhejiang Cancer Hospital from January 2017 to December 2020. Breast scanning was performed using a GE LightSpeed-RT CT simulator or a Philips large-aperture CT simulator. The CT layer thickness was 5 mm and the pixel matrix had a size of 512 × 512. After scanning, the PTV, heart, ipsilateral lung, contralateral lung, and spinal cord were delineated by senior physicians with 10 years of expertise. No regions of interest overlapped with each other. Each patient received breast-conserving radiotherapy on the left side, where the internal breast region and cervical lymph nodes were not subjected to radiation. Actual treatment was carried out using volumetric-modulated arc therapy (VMAT) techniques with 6-MV double-arc X-ray. Also, the start and stop angles of the X-ray gantry were tangent to the irradiated breast. The prescribed dose was 5000 cGy spread over 25 fractions, and scaled to cover 95% of the PTV for all cases. The ROI delineation and treatment planning were completed using the RayStation radiotherapy planning system (Version 4.5, RaySearch Laboratories AB, Sweden). All plans were optimized further using a trial-and-error process to conform to the as-low-as-reasonably achievable principle for normal tissues. Moreover, all plans were reviewed by two experienced dosimetrists and one senior oncologist.

### Data preprocessing

For each patient in the training set, the ROI structures and the corresponding dose distribution were converted from the Digital Imaging and Communications in Medicine (DICOM) format to matrices in Python. In this conversion process, each voxel was assigned a specific value based on the ROI type. Specifically, the voxels in the ROIs of the PTV, the ipsilateral lung, the heart, the contralateral lung, the spinal cord, and other tissues were assigned values of 1.000, 0.833, 0.667, 0.500, 0.333, and 0.167, respectively, whereas voxels outside of the body contour were assigned a value of 0. For each case, every ROI matrix retained 64 slices in the head–foot direction, and all PTV was contained within the 64 slices. In the transverse section, each slice matrix was bilinearly interpolated into a size of 256 × 256 to reduce the computational cost. The dose distribution matrices were derived from the DICOM data, and each dose value was scaled to be in the range [0, 1] through division by 6000 cGy. For each case, the dose matrix was bilinearly interpolated into a size of 64 × 256 × 256, which corresponds to the ROI image matrix size.

### Fully convolutional networks

**Fig. 5:**
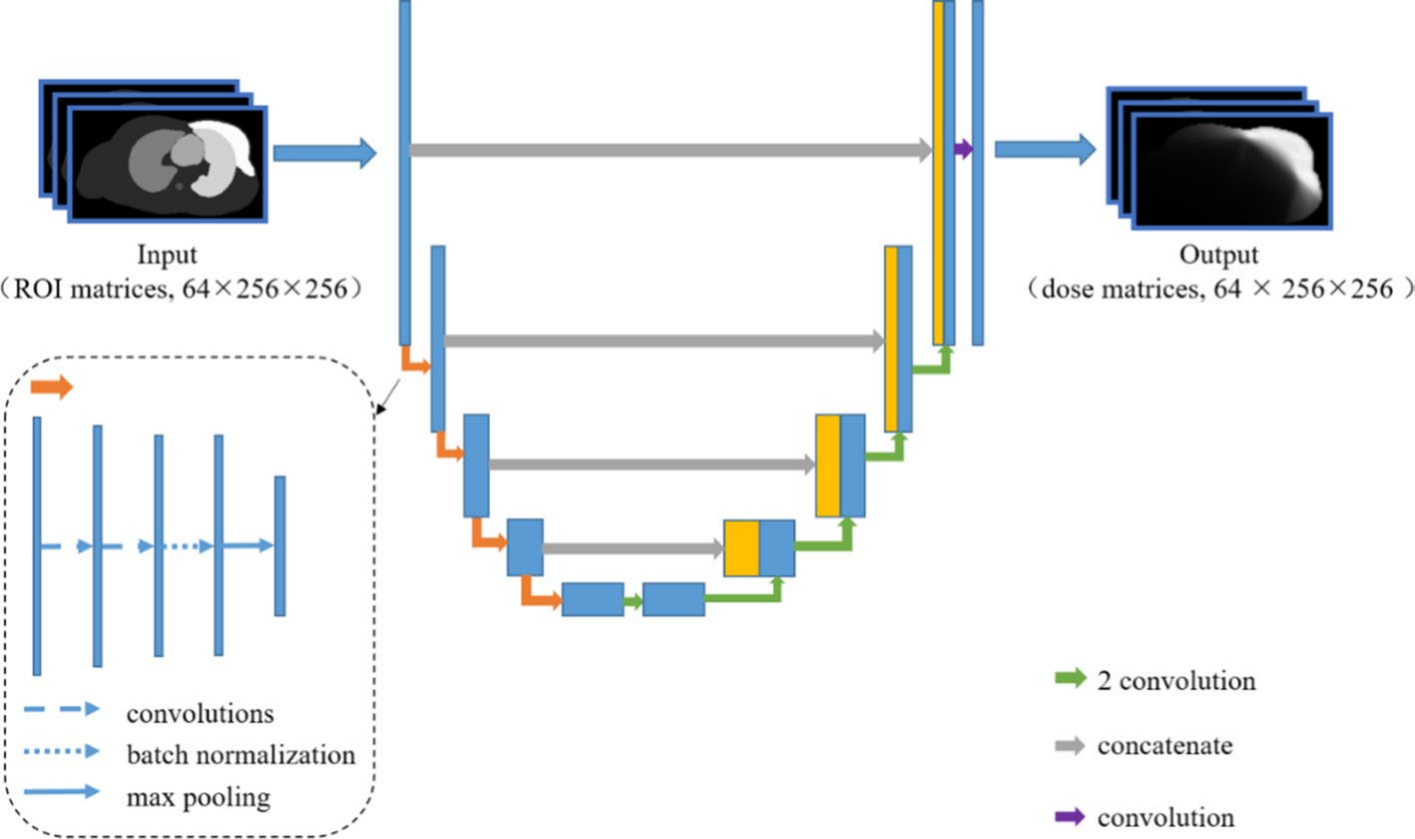
The architecture of the proposed deep network architecture for radiotherapy dose prediction

The U-Net FCN architecture was used to predict dose distributions in breast cancer. This choice is motivated by earlier successful applications of this architecture in medical image segmentation as well as dose prediction for head and neck cancer, prostate cancer and breast cancer [20, 22, 23]. The U-Net model used in this study has a total of 10 layers, with a network input of 3-dimensional 64 × 256 × 256 ROI matrices with 1 channel. Each of the first 5 layers is a down-sampling layer that contains two 3 × 3 × 3 convolution modules with valid padding, followed by a rectified linear unit (ReLU), one batch normalization module, and one 2 × 2 × 2 maximum pooling operation. The 6th, 7th, 8th, and 9th layers are up-sampling layers. Each of these layers has one concatenation module, two 3 × 3 × 3 convolution modules with valid padding, followed by a ReLU module, and one batch normalization module. Finally, the 10th layer has a 1 × 1 × 1 convolution module followed by a linear activation function for output, and the output of this layer is the dose distribution matrix. The overall architecture of the proposed network is shown in Fig. 5.

### Sharp loss

The sharp loss function is introduced in this work to better address the problem of data imbalance for dose distribution prediction in radiotherapy. Indeed, training data for this problem typically show an extreme imbalance between the high-dose volume (> 500 cGy) and the low-dose volume (< 500 cGy). The sharp loss function is derived from the MSE loss:

2 $${\text{MSE}} = \frac{1}{n}\mathop \sum \limits_{i = 1}^{n} \left( {Dp_{i} - D_{i} } \right)^{2} ,$$

where $n$ was the total number of voxels, $Dp_{i}$ is the predicted dose for voxel *i*, and $D_{i}$ is the ground-truth dose for voxel *i*.

Figure 3 shows a plot of the MSE loss (in blue) as a function of the ground-truth dose value. One remarkable property of this loss is that even a voxel dose that is close to 0 incurs a loss with a non-trivial magnitude. When small loss values are summed over a large number of low-dose voxels, the clinically more relevant high-dose voxels will be overwhelmed.

The following experiments stress that remark: the large number of low-dose voxels encountered during training of the U-Net architecture dominates the MSE loss. In fact, the low-dose voxels, especially those outside the body, comprise the majority of the MSE loss and hence dominate the loss function gradient. In order to redirect the network optimization process to focus on the more relevant higher-dose voxels, the sigmoid function is employed to reduce the weight of the low-dose voxels in the network loss. The sigmoid function is mathematically defined as:

3 $$S\left( x \right) = \frac{1}{{1 + e^{ - \gamma x} }}, \gamma = 1.$$

**Fig. 6:**
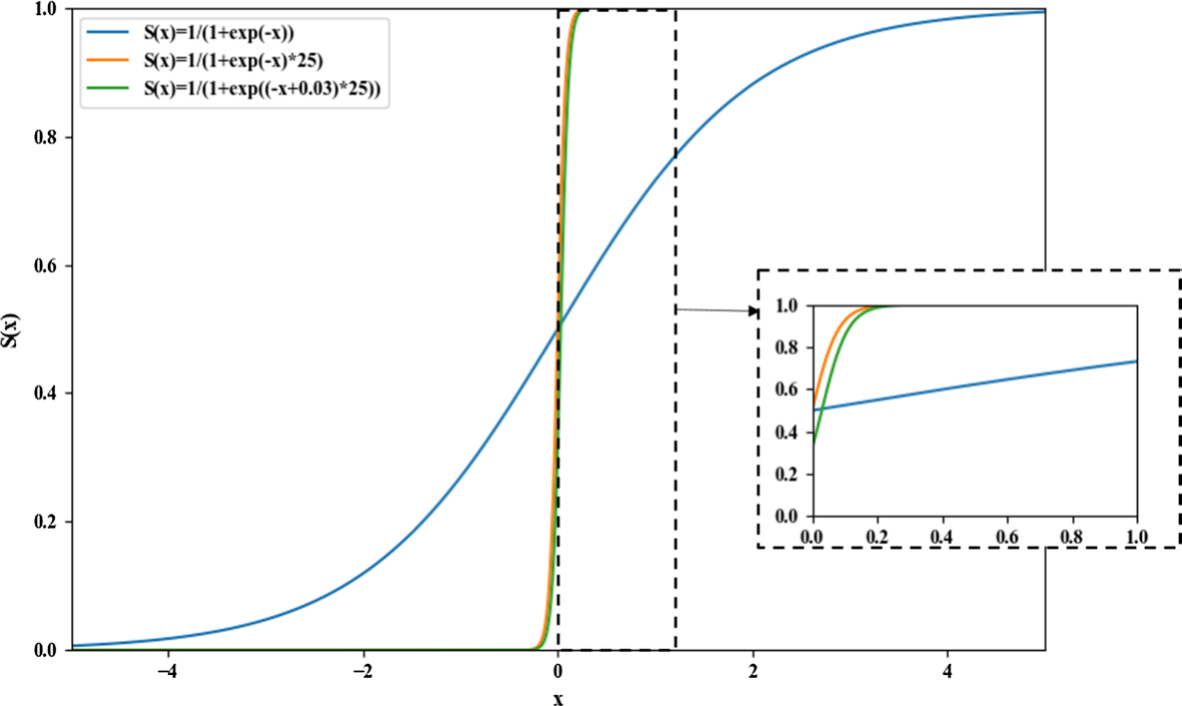
The original sigmoid function (in blue) and its two variants (in orange and green, respectively)

Figure 6 shows a plot of this function (in blue). Because the sigmoid function is smooth and easily differentiable, it is widely used in deep learning to map real numbers into the unit interval [0, 1]. In this work, the definition of the sigmoid function was modified in terms of two aspects. First, since the predicted dose values were scaled to be in the range [0, 1], the γ factor was allowed to exceed the unit value in order to increase the loss gradient in the dose value range, especially for low-dose values. A modified sigmoid function with $\gamma = 25$ is shown as the orange curve in Fig. 6. A larger $\gamma$ value resulted in a steeper function behavior in the low-dose region. Second, the loss curve was moved toward the positive direction of the *x* axis by subtracting 0.03 from *x* to keep the ground-truth dose values positive. This function modification can lead to more efficient exploitation of the gradient regions of the sigmoid function. This modified function is shown in Fig. 6 as the green curve.

As an alternative to the MSE loss, we propose in this study to reshape the loss function to assign lower weights to low-dose voxels, and thus focus the network training on the high-dose voxels. The modified sigmoid function introduced above was proposed as a modulating factor for the MSE loss. We name the modulated loss function the ‘sharp loss’, which is defined as:

4 $${\text{SL}} = \frac{1}{n}\mathop \sum \limits_{i = 1}^{n} \frac{1}{{1 + e^{{ - \left( {D_{i} - 0.03} \right) \times \gamma }} }}\left( {Dp_{i} - D_{i} } \right)^{2} ,$$

where $\gamma \ge 0$ is a tunable parameter. The factor $1/\left( {1 + e^{{ - \left( {D_{i} - 0.03} \right) \times \gamma }} } \right)$ was derived from the sigmoid function as explained above. Figure 3 shows plots of the sharp loss function for several $\gamma$ values. For this proposed loss function, when a ground-truth dose value is close to 0, the loss for this voxel is assigned a low weight and approaches 0. Since most of the zero-dose voxels are outside the body, the prediction precision of the low-dose voxels will not cause large errors inside the body. In the following experiments, we analyzed the dose prediction performance with the sharp loss function for $\gamma = 1, 25, 50, 100, 250,$ and $500$.

### Network model training

Ten breast cancer cases were randomly selected from the 110 available cases to form the test set, while the remaining 100 cases comprised the training set. The training data included a total of 100 three-dimensional ROI arrays and their corresponding dose matrices. The ROI arrays were used as the input data while the corresponding dose matrices were employed as the output data for prediction model training. A tenfold cross-validation scheme was employed. The network construction was implemented in the Python-based application programming interface of Keras (https://keras.io/). The Adam algorithm was used for loss function minimization. The batch size was set to 2, and the initial learning rate was set to 0.001. Also, when the validation loss did not decrease in 5 iterations, the learning rate was attenuated at a rate of 0.1 until it reached ${10}^{-7}$. The training was carried out using two Intel Xeon Gold 5118 CPUs @ 2.30 GHz, and one NVIDIA TITAN RTX PC with a 192-GB RAM. After network training, the ROI arrays of the test set were fed into the trained model, and the corresponding dose prediction matrices were obtained.

### Performance validation

The performance of the proposed system was validated on the 10 selected test cases. MAD between the ground-truth and predicted dose distributions was used to evaluate the prediction performance. The MAD performance metric is defined as:

5 $${\text{MAD}} = \frac{1}{n}\mathop \sum \limits_{i = 1}^{n} \left( {\left| {Dp_{i} - D_{i} } \right|} \right),$$

where *n* is the total number of voxels inside the evaluated area, while $Dp_{i}$ and $D_{i}$ are the ground-truth and predicted dose values for voxel *i*.

To assess the statistical significance of the prediction results obtained by different loss functions, a Wilcoxon signed-rank test was performed with a significance level of *p* = 0.05 [34]. Statistical analyses were conducted using SPSS v19 (IBM, Armonk, NY, USA).

## Data Availability

The datasets used and/or analyzed during the current study are available from the corresponding author on reasonable request.

## Abbreviations

OAR: Organs at risk
DVH: Dose–volume histograms
CT: Computed tomography
ROI: Regions of interest
CNN: Convolutional neural networks
FCN: Fully convolutional networks
MSE: Mean squared error
MAD: Mean absolute difference
PTV: Planning target volumes
HI: Homogeneity index
CI: Conformation index
VMAT: Volumetric-modulated arc therapy
DICOM: Digital Imaging and Communications in Medicine
ReLU: Rectified linear unit
